# Direct information retrieval after 3D reconstruction in grating-based X-ray phase-contrast computed tomography

**DOI:** 10.1107/S1600577518008019

**Published:** 2018-06-27

**Authors:** Zhao Wu, Kun Gao, Zhili Wang, Chenxi Wei, Faiz Wali, Guibin Zan, Wenbin Wei, Peiping Zhu, Yangchao Tian

**Affiliations:** a National Synchrotron Radiation Laboratory, University of Science and Technology of China, Hefei, Anhui 230029, People’s Republic of China; b School of Electronic Science and Applied Physics, Hefei University of Technology, Hefei, Anhui 230009, People’s Republic of China; c Institute of High-Energy Physics, Chinese Academy of Sciences, Beijing 100049, People’s Republic of China

**Keywords:** direct information retrieval method, grating-based phase-contrast imaging, differential phase-contrast imaging, computed tomography

## Abstract

Grating-based X-ray differential phase-contrast imaging has attracted a great amount of attention and has been considered as a potential imaging method in clinical medicine because of its compatibility with the traditional X-ray tube source and the possibility of a large field of view. Moreover, phase-contrast computed tomography provides three-dimensional phase-contrast visualization. Generally, two-dimensional information retrieval performed on every projection is required prior to three-dimensional reconstruction in phase-contrast computed tomography. In this paper, a three-dimensional information retrieval method to separate absorption and phase information directly from two reconstructed images is derived. Theoretical derivations together with numerical simulations have been performed to confirm the feasibility and veracity of the proposed method. The advantages and limitations compared with the reverse projection method are also discussed. Owing to the reduced data size and the absence of a logarithm operation, the computational time for information retrieval is shortened by the proposed method. In addition, the hybrid three-dimensional images of absorption and phase information were reconstructed using an absorption reconstruction algorithm, hence the existing data pre-processing methods and iterative reconstruction algorithms in absorption reconstruction may be utilized in phase reconstruction immediately.

## Abstract 2

The theoretical framework of the direct information retrieval method is presented in phase-contrast computed tomography. Numerical simulations are also performed, which reveal that the proposed method provides comparable results of the reverse projection method with short computational time. Furthermore, the compatibility with the existing data preprocessing methods and iterative reconstruction algorithms is discussed.

## 1. Introduction   

X-ray phase-contrast imaging provides information about an object that would be otherwise inaccessible (Nugent *et al.*, 1996 ▸; Fitzgerald, 2000 ▸) using conventional attenuation-based X-ray imaging. Along with improved X-ray sources and optical components over the last two decades, X-ray phase-contrast imaging has experienced a great evolution (Wilkins *et al.*, 1996 ▸; Momose *et al.*, 1996 ▸; Zhou & Brahme, 2008 ▸; Pfeiffer *et al.*, 2008 ▸; Tapfer *et al.*, 2011 ▸; Thuering *et al.*, 2011 ▸; Bravin *et al.*, 2013 ▸; Wu *et al.*, 2015 ▸), especially with the introduction of grating-based X-ray phase-contrast imaging (GBPCI), which included Talbot interferometers (Momose *et al.*, 2003 ▸; Weitkamp *et al.*, 2005 ▸; Xi *et al.*, 2012 ▸), Talbot–Lau interferometers (Pfeiffer *et al.*, 2006 ▸) and non-interferometric grating-based imaging (Huang *et al.*, 2009 ▸). Owing to its compatibility with conventional X-ray sources and the possibility of a large field of view, GBPCI has been considered as a potential imaging method in clinical medicine. In addition, phase-contrast computed tomography (PCCT) provides three-dimensional phase-contrast visualization from the retrieved two-dimensional refraction angle images at numerous tomographic viewing angles using a reconstruction algorithm. Generally, two-dimensional information retrieval is required prior to independent and discriminating absorption and phase three-dimensional reconstruction. The existing data pre-processing methods and computed tomography (CT) algorithms in absorption reconstruction cannot be employed directly in phase reconstruction.

Recently, many highly efficient and ingenious information retrieval techniques (Zanette *et al.*, 2012 ▸; Wang *et al.*, 2014 ▸; Zhu *et al.*, 2010 ▸; Yang *et al.*, 2016 ▸) have been put forward to improve the performance of grating-based PCCT in terms of acquisition time and radiation dose. Following previous research in diffraction enhanced imaging (Zhu *et al.*, 2006 ▸; Wang *et al.*, 2007 ▸), Zhu *et al.* (2010 ▸) presented a fast and low-dose information retrieval method known as the reverse projection (RP) method. The phase information collapse phenomenon (Raupach & Flohr, 2011 ▸) can be avoided in this method; however, information retrieval still needs to be performed before three-dimensional reconstruction. Afterwards, Diemoz *et al.* (2011 ▸) proposed a simplified method, which obtained three-dimensional information without information retrieval. However, the displayed three-dimensional mixed image, containing both absorption and phase information, is not quantitative and is unable to take full advantage of the high contrast of phase-contrast images. Brendel *et al.* (2016 ▸) achieved three types of information tomography using iterative reconstruction without information retrieval.

Inspired by these methods, we present a direct three-dimensional information retrieval method, where three-dimensional reconstruction can be achieved using an absorption CT algorithm before information retrieval. Therefore, the existing data pre-processing methods and iterative reconstruction algorithms in absorption reconstruction may be introduced to phase reconstruction immediately. In addition, the computational time of the information retrieval procedure is shortened, owing to the reduced data size and the absence of the logarithm operation. Theoretical derivations and numerical simulations have been carried out to confirm its feasibility and validity.

## 2. Grating-based phase-contrast imaging setup and three-dimensional information retrieval method   

### 2.1. Grating-based phase-contrast imaging setup   

**Figure 1:**
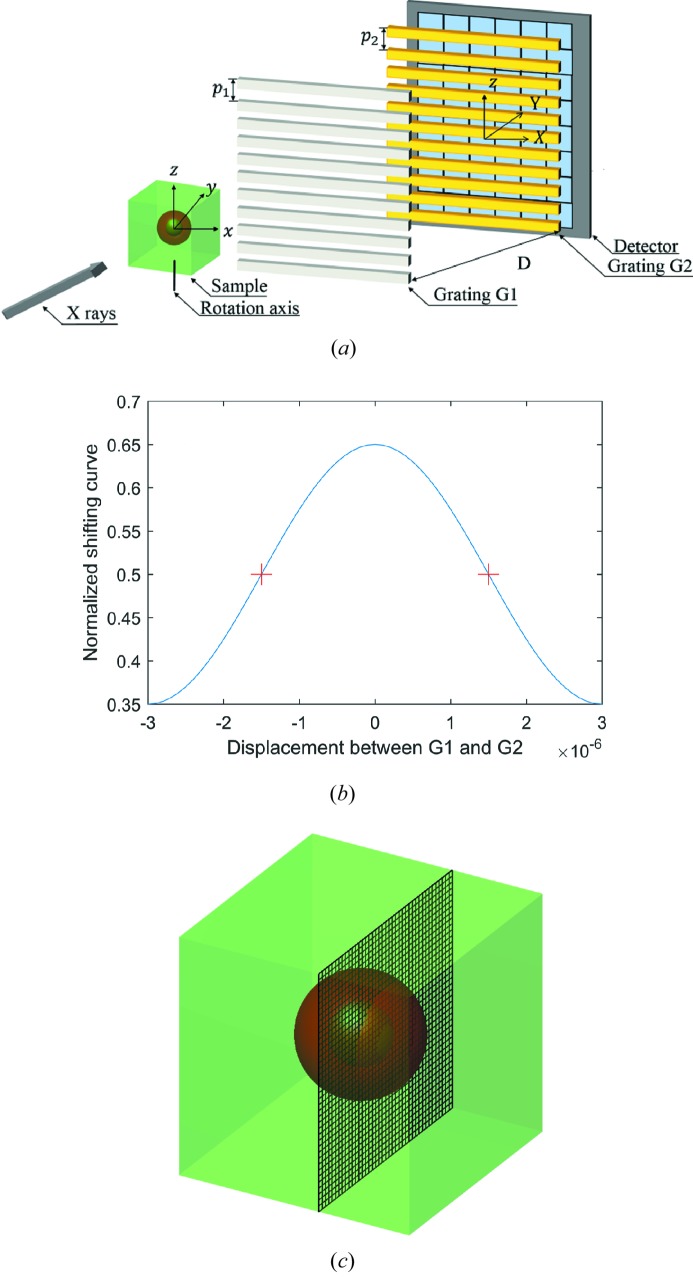
(*a*) Schematic diagram of the grating-based X-ray Talbot interferometer; (*b*) the normalized shifting curve with a visibility of 0.3 and (*c*) the sample in the numerical experiments, a cube of polyethyl­ene 2.55 cm × 2.55 cm × 2.55 cm in size. The inner structure of the sample, rendered in red, is a spherical shell of polycarbonate with an inner diameter of 0.64 cm and an external diameter of 1.28 cm.

For simplicity, we use a parallel-beam Talbot interferometer, for example, to interpret and certify the direct three-dimensional information retrieval method. As described in Fig. 1 ▸(*a*), a Talbot interferometer consists of a phase-shift grating (G1), an absorption grating (G2) and a detector. Here, the system rotation axis is vertical with respect to the grating lines so that the physical quantities are rotation invariants (Zhu *et al.*, 2005 ▸). The Cartesian coordinate system (*x*, *y*, *z*) and (*X*, *Y*, *z*) are, respectively, the reference frame of the sample and the imaging system. The detected photon number at pixel (*X*, *z*), where *X* and *z* are integers, can be expressed as follows with negligible scattering of the object (Zhu *et al.*, 2010 ▸):
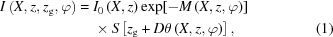
where *z*_g_ is the displacement along the direction perpendicular to the beam axis and the grating lines between G1 and G2, φ is the tomographic viewing angle and *I*_0_ is the detected photon number without the gratings and sample in the light path. The function *S*(*z*_g_) describes the normalized background shifting curve (SC). *M* represents the attenuation term, which can be formulated by the integral of the absorption coefficient of the object along the light path:
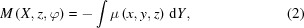
the fractional Talbot distance *D* is the distance between G1 and G2, and 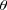 is the refraction angle induced by the object, associated with the real-part decrement δ of the refractive index by
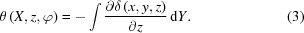



The normalized background SC *S*(*z*_g_) shown in Fig. 1 ▸(*b*) can be plotted by recording the photon number with the increase of *z*_g_ when the object is absent in the light path.

### 2.2. Three-dimensional information retrieval method   

Carrying out the logarithmic operation on both sides of equation (1) yields:
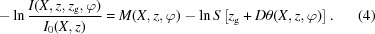
It is worth noting that the logarithm of the SC can be well approximated to the first-order Taylor expansion around up-slope and down-slope positions indicated in Fig. 1 ▸(*b*),
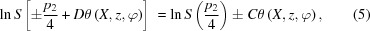
where 
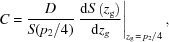
the axial symmetry of the SC, has been considered in the derivation. Substituting equations (2), (3) and (5) into equation (4) gives
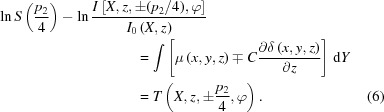



The hybrid three-dimensional data of the absorption and phase information can be reconstructed by absorption CT algorithms. A conventional filtered backprojection (FBP) method (Huang *et al.*, 2006 ▸) is employed for reconstruction in equation (7):
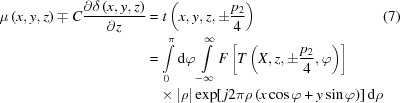
where *F* and 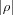 stand for the Fourier transform and the absolute value of the frequency, respectively. Then, the three-dimensional absorption coefficient and phase-contrast data are obtained by:
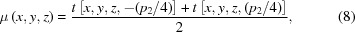


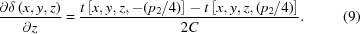



It can be found from equations (8) and (9) that the three-dimensional absorption and phase-contrast images are retrieved directly from two three-dimensional images reconstructed by an absorption CT algorithm. Thus the two-dimensional information retrieval procedures at every tomographic viewing angle are totally abandoned and a direct three-dimensional information retrieval method can be accessible in theory. Furthermore, we predict that this method has the potential to make a direct introduction of the established data pre-processing techniques and other advanced CT algorithms in absorption reconstruction to phase reconstruction.

## 3. Numerical simulations   

Numerical simulations have been performed to validate the feasibility and veracity of the proposed method. Perfect gratings and alignment are assumed in the simulations. The energy was designed at 25 keV and the pitches of the phase shift (π/2) grating and analyzer grating were 6 µm. We placed G2 at the first fractional Talbot distance where *D* = 0.3629 m downstream from G1 along the beam axis. The detector with pixel size of 100 µm was located closely after the grating G2. The visibility of the background SC was fixed at 0.3, referring to the reported value in a laboratory experiment (Zhu *et al.*, 2010). The incoming photon number *I*_0_ was 10000 per pixel and Poisson noise was introduced. The simulated phantom shown in Fig. 1(*c*) is a cube of polyethylene with 255 × 255 × 255  volume elements. The inner structure consists of a spherical shell of polycarbonate with an inner diameter of 0.64 cm and an external diameter of 1.28 cm. The attenuation coefficients and the real-part decrements of the refractive indexes of the materials are listed in Table 1 ▸ (refer to the CSIRO website, https://www.ts-imaging.net/Services/Simple/ICUtilXdata.aspx).

In the simulations, two sets of 360 projection images have been collected evenly around a circle, when the grating G2 was positioned at the up-slope and down-slope positions. We obtained three-dimensional absorption and phase-contrast images by the RP method and the proposed method. In the RP method, the two-dimensional phase and absorption information were separated first and then reconstructed with different CT algorithms independently. In the proposed method, two hybrid three-dimensional images were reconstructed by an absorption CT algorithm and then the three-dimensional information was separated using equations (8) and (9).

**Figure 2:**
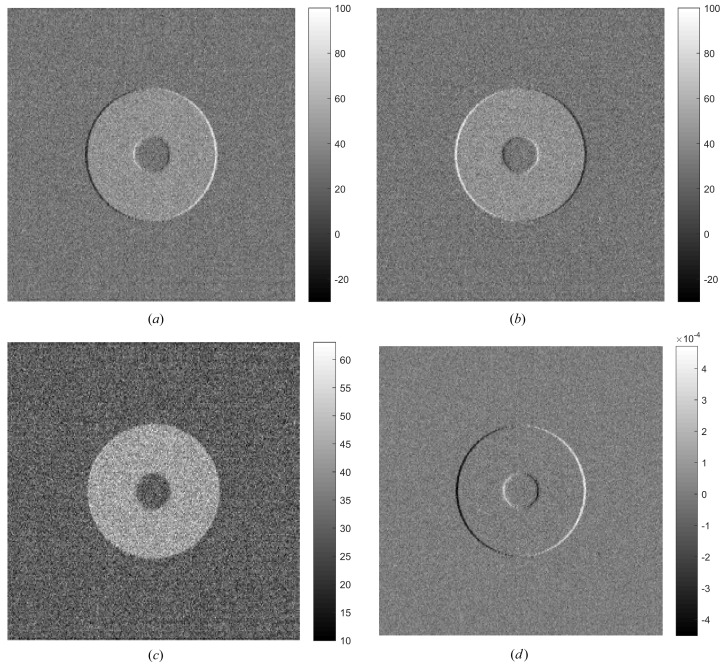
Results of the direct three-dimensional information retrieval method; coronal slices at the black grid plane are depicted in Fig. 1 ▸(*c*). Reconstructed images at the up-slope (*a*) and down-slope (*b*); retrieved absorption image (*c*) and differential phase-contrast image (*d*).

The coronal slices in the black-grid plane depicted in Fig. 1 ▸(*c*) of the reconstructed mixed images at the up-slope and down-slope positions are illustrated in Figs. 2 ▸(*a*) and 2(*b*). We can see that they are almost identical with the exception of the boundary of the annulus where the phase signal is non-zero. This is because both images contain the same attenuation coefficient and the opposite derivative of the real-part decrement of the refractive index as revealed in equation (6). Using the information retrieval formulae (8) and (9), the absorption and phase-contrast images in Figs. 2(*c*) and 2(*d*) can be retrieved directly.

**Figure 3:**
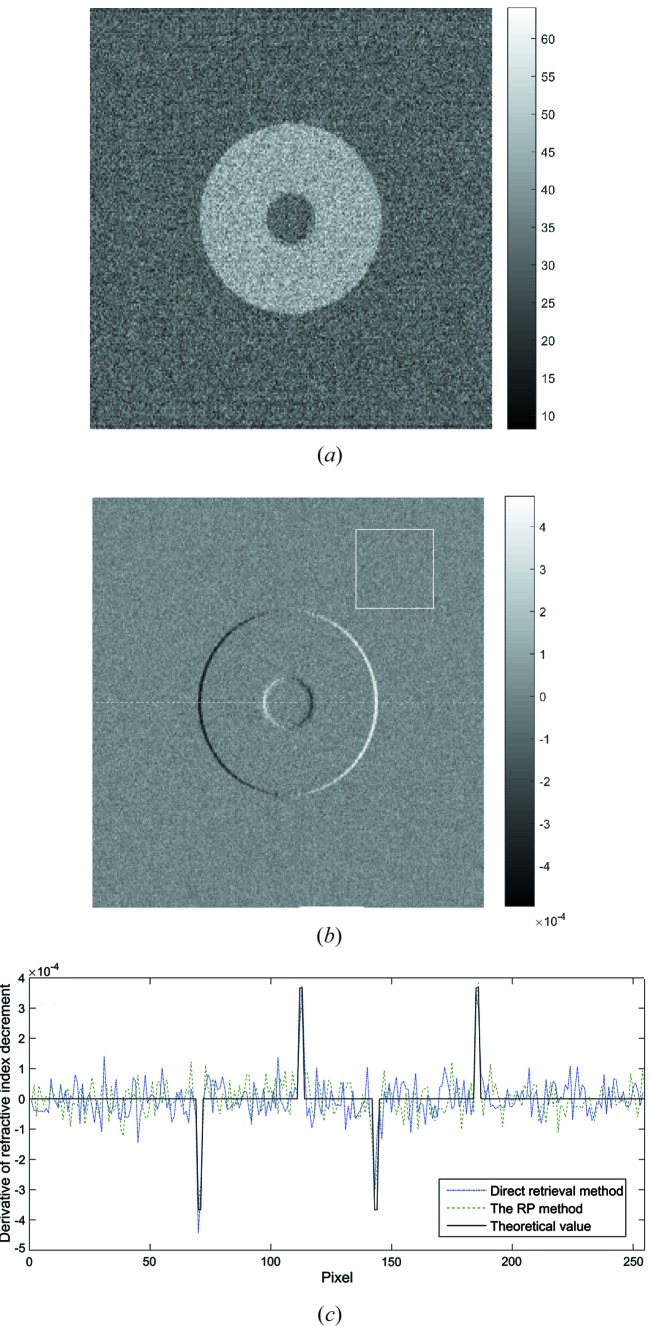
(*a*) The corresponding absorption image and (*b*) the differential phase contrast image by the RP method. The pixels in the white square in (*b*) were selected to evaluate the noise variances of the reconstricted slices. (*c*) The profiles of the theoretical and retrieved phase information obtained by the two methods at the white dashed line plotted in (*b*).

As illustrated in Figs. 3(*a*)–3(*b*), the reconstructed absorption and phase-contrast images by the RP method are almost identical to their counterparts obtained by the direct retrieval method. In Fig. 3 ▸(*c*), we present the profiles of the theoretical- and retrieved-phase information, obtained by the RP method and the direct retrieval method, respectively, at the white dashed line plotted in Fig. 3 ▸(*b*). The good agreement between the two methods confirms the feasibility and veracity of the proposed method. The similar noise variances of 50 × 50 pixels in the white square in Fig. 3 ▸(*b*) (1.81 × 10^−9^ and 1.83 × 10^−9^) confirm the equivalence of the direct retrieval method and the RP method.

The simulations were repeated five times to compare the computational complexity between the RP method and the proposed method. The computational time is recorded in Table 2 ▸, where DIR and RP stand for the presented direct information method and the reverse projection method, respectively. It reveals that the time for the information retrieval is significantly shortened by the proposed method. This may be explained by the reduced data size, from 360 × 363 × 255 pixels in the RP method to 255 × 255 × 255 pixels in the DIR method, and the absent logarithm operation. It is worth noting that the total computing efficiency becomes significant when the ratio between the tomographic viewing angle and the number of pixels in the width increases. Moreover, the computational time of CT reconstruction executed on graphic processing units (GPUs) or field-programmable gate arrays (FPGAs) will be sharply reduced, similar to the speedups of 26.9 for FPGAs in Choi & Cong (2016 ▸) and 22 for GPUs and 35.8 for FPGAs in Després & Jia (2017 ▸). Assuming the computational time of CT reconstruction is speeded up 20 times, we can predict that more than one-third of the total computing time of the RP method will be saved by employing the DIR method in medical 16-slice CT data with a size of 2880 × 816 × 16 pixels.

## 4. Discussions   

In this paper, we derive the direct three-dimensional information retrieval method in parallel beam geometry, which can also be employed in the case of fan beam geometry. Taking equiangular fan beam geometry as an example, we introduce a polar coordinate (*r*, α, *z*) to describe the imaging system. When the sample is placed downstream of the grating G1, and the distance between sample and detector is *R*, the detected photon number is (Wu *et al.*, 2013 ▸):
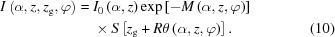
The absorption term and refraction angle can be expressed as
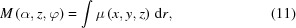


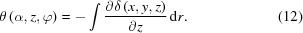



Further derivations can be completed by referring to equations (4)–(9), replacing equation (7) with a fan beam FBP reconstruction algorithm (Wu, 2017 ▸). One can also complete the derivations for the case where the sample is in front of the grating G1, referring to our previous paper (Wu *et al.*, 2013 ▸). It should be noted that this method is not suitable for cone beam geometry because of the change in the direction of partial derivatives of δ at the plane *z* ≠ 0.

Compared with the previous method, the proposed method has two advantages: computational efficiency, which has been discussed in §3; and compatibility with the existing data pre-processing methods, such as the ring correction method (Kim *et al.*, 2014 ▸) and iterative reconstruction algorithms (Arcadu *et al.*, 2017 ▸; Wang *et al.*, 2017 ▸) in conventional absorption-based computed tomography. In the line-ratio ring artefacts correction method, the sensitivity difference between adjacent detector elements can be extracted from the ratios of the sum at the up-slope and down-slope of adjacent detectors along all projection angles. In contrast, the information is derived from values at two different detector positions in the scanning mode mentioned by Zhu *et al.* (2010 ▸), so the line-ratio ring artefacts correction method is invalid in that case. Furthermore, taking the physical quantity of 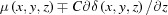 as a whole, the absorption-based iterative reconstruction algorithms are directly generalized to phase-contrast tomography due to the rotation invariance. Hence, interior tomography and short-scan tomography in phase-contrast imaging may be handled immediately.

We would like to point out several limitations of the proposed method:

(1) Perfect gratings and alignment are required, which limits the field of view or reduces imaging performance. With the development of grating fabrication and large periodic grating-based non-interferometric imaging (Huang *et al.*, 2009 ▸), these problems may be mitigated.

(2) The proposed direct information retrieval method is only discussed for the case where the object is free of the scattering information. As a matter of fact, if the object possesses high scattering information but weak phase information, a direct three-dimensional information retrieval method can also be carried out at peak and valley positions for attenuation and scattering information.

(3) The retrieved phase information is the derivative of the real-part decrement of the refractive index. The real-part decrement of the refractive index free of stripe artefacts can be recovered with an iterative method proposed by Thüring *et al.* (2011 ▸).

(4) A two-step scanning mode is required in the presented method; however, there is only one step in the RP method. Recently, a newly designed grating was introduced in an applicable imaging method without mechanical phase stepping (Wei *et al.*, 2017 ▸). The proposed method can also realise single-shot imaging with this grating.

## 5. Conclusions   

In summary, we have presented a direct three-dimensional information retrieval method in grating-based X-ray differential phase-contrast computed tomography. This method obtains the absorption coefficient and the derivative of the real-part decrement of the refractive index immediately from two three-dimensional reconstructed images. Compared with the previous method, the proposed method improves the computational efficiency with comparative image quality and introduces the existing data pre-processing methods and the iterative reconstruction algorithms in absorption reconstruction to phase reconstruction directly. The presented method also lends itself to other X-ray differential phase-contrast imaging, such as analyzer-based imaging and edge illumination.

## Figures and Tables

**Table 1:** Attenuation coefficients and real part decrements of polyethyl­ene and polycarbonate

	Colour in Fig. 1 ▸(*c*)	Attenuation coefficients μ (m^−1^)	Real-part decrements δ
Polyethyl­ene	Green	29.77	3.4977 × 10^−7^
Polycarbonate	Red	43.14	4.2312 × 10^−7^

**Table 2:** Comparison of the computational time between the three-dimensional direct information retrieval method and the reverse projection method

	Information retrieval			CT			Total		
	DIR (s)	RP (s)	DIR/RP†	DIR (s)	RP (s)	DIR/RP†	DIR (s)	RP (s)	DIR/RP†
1	0.17	0.57	0.29	19.52	19.18	1.00	19.69	19.75	0.98
2	0.16	0.57		19.50	19.68		19.66	20.25	
3	0.17	0.57		19.49	19.89		19.66	20.46	
4	0.16	0.57		19.58	19.64		19.74	20.21	
5	0.18	0.58		19.91	19.22		20.09	19.80	

† The ratio is between the average values.
